# Quasipolynomial Computation of Nested Fixpoints

**DOI:** 10.1007/978-3-030-72016-2_3

**Published:** 2021-03-01

**Authors:** Daniel Hausmann, Lutz Schröder

**Affiliations:** 8 Eindhoven University of Technology, Eindhoven, The Netherlands; 9 Aalborg University, Aalborg East, Denmark; Friedrich-Alexander-Universität Erlangen-Nürnberg, Erlangen, Germany

**Keywords:** Fixpoint theory, model checking, satisfiability checking, parity games, energy games, $\mu$-calculus

## Abstract

It is well-known that the winning region of a parity game with *n* nodes and *k* priorities can be computed as a *k*-nested fixpoint of a suitable function; straightforward computation of this nested fixpoint requires $\mathcal {O}(n^{\frac{k}{2}})$ iterations of the function. Calude et al.’s recent quasipolynomial-time parity game solving algorithm essentially shows how to compute the same fixpoint in only quasipolynomially many iterations by reducing parity games to quasipolynomially sized safety games. Universal graphs have been used to modularize this transformation of parity games to equivalent safety games that are obtained by combining the original game with a universal graph. We show that this approach naturally generalizes to the computation of solutions of systems of *any* fixpoint equations over finite lattices; hence, the solution of fixpoint equation systems can be computed by quasipolynomially many iterations of the equations. We present applications to modal fixpoint logics and games beyond relational semantics. For instance, the model checking problems for the energy $\mu$-calculus, finite latticed $\mu$-calculi, and the graded and the (two-valued) probabilistic $\mu$-calculus – with numbers coded in binary – can be solved via nested fixpoints of functions that differ substantially from the function for parity games but still can be computed in quasipolynomial time; our result hence implies that model checking for these $\mu$-calculi is in $\textsc {QP}$. Moreover, we improve the exponent in known exponential bounds on satisfiability checking.

## 1 Introduction

Fixpoints are pervasive in computer science, governing large portions of recursion theory, concurrency theory, logic, and game theory. One famous example are parity games, which are central, e.g., to networks and infinite processes [5], tree automata [43], and $\mu$-calculus model checking [17]. Winning regions in parity games can be expressed as nested fixpoints of particular set functions (e.g. [8,16]). In recent breakthrough work on the solution of parity games in quasipolynomial

⋆ Work forms part of the DFG-funded project CoMoC (SCHR 1118/15-1, MI 717/7-1).

time, Calude et al. [9] essentially show how to compute this particular fixpoint in quasipolynomial time, that is, in time $2^{\mathcal{O}((\log n)^c)}$ for some constant $c$. Subsequently, it has been shown [13,14,28] that universal graphs (that is, even graphs into which every even graph of a certain size embeds by a graph morphism) can be used to transform parity games to equivalent safety games obtained by pairing the original game with a universal graph; the size of these safety games is determined by the size of the employed universal graphs and it has been shown [13,14] that there are universal graphs of quasipolynomial size. This yields a uniform algorithm for solving parity games to which all currently known quasipolynomial algorithms for parity games have been shown to instantiate using appropriately defined universal graphs [13, 14].

Briefly, our contribution in the present work is to show that the method of using universal graphs to solve parity games generalizes to the computation of nested fixpoints of arbitrary functions over finite lattices. That is, given functions $f_i : \mathcal{P}(U)^{k+1} \to \mathcal{P}(U)$, $0 \leq i \leq k$ on a finite lattice $U$, we give an algorithm that uses universal graphs to compute the solutions of systems of equations

$$X_i =_{\eta_i} f_i(X_0, \ldots, X_k) \qquad\qquad 0 \leq i \leq k$$

where $\eta_i = \mathsf{GFP}$ (greatest fixpoint) or $\eta_i = \mathsf{LFP}$ (least fixpoint). Since there are universal graphs of quasipolynomial size, the algorithm requires only quasipolynomially many iterations of the functions $f_i$ and hence runs in quasipolynomial time, provided that all $f_i$ are computable in quasipolynomial time. While it seems plausible that this time bound may also be obtained by translating equation systems to equivalent standard parity games by emulating Turing machines to encode the functions $f_i$ as Boolean circuits (leading to many additional states but avoiding exponential blowup during the process), we emphasize that the main point of our result is not so much the ensuing time bound but rather the insight that universal graphs and hence many algorithms for parity games can be used on a much more general level which yields a precise (and relatively low) quasipolynomial bound on the number of function calls that are required to obtain solutions of fixpoint equation systems.

In more detail, the method of Calude et al. can be described as annotating nodes of a parity game with histories of quasipolynomial size and then solving this annotated game, but with a safety winning condition instead of the much more involved parity winning condition. It has been shown that these histories can be seen as nodes in universal graphs, in a more general reduction of parity games to safety games in which nodes from the parity game are annotated with nodes from a universal graph. This method has also been described as pairing *separating automata* with safety games [14]. It has been shown [13,14] that there are exponentially sized universal graphs (essentially yielding the basis for e.g. the fixpoint iteration algorithm [8] or the small progress measures algorithm [27]) and quasipolynomially sized universal graphs (corresponding, e.g., to the succinct progress measure algorithm [28], or to the recent quasipolynomial variant of Zielonka's algorithm [38]).

Hasuo et al. [22], and more generally, Baldan et al. [4] show that nested fixpoints in highly general settings can be computed by a technique based on progress measures, implicitly using exponentially sized universal graphs, obtaining an exponential bound on the number of iterations. Our technique is based on showing that one can make explicit use of universal graphs, correspondingly obtaining a quasipolynomial upper bound on the number of iterations. In both cases, computation of the nested fixpoint is reduced to a single (least or greatest depending on exact formulation) fixpoint of a function that extends the given set function to keep track of the exponential and quasipolynomial histories, respectively, in analogy to the previous reduction of parity games to safety games. Our central result can then be phrased as saying that the method of transforming parity conditions to safety conditions using universal graphs generalizes from solving parity games to solving systems of equations that use arbitrary functions over finite lattices. We use *fixpoint games* [4, 42] to obtain the crucial result that the solutions of equation systems have history-free witnesses, in analogy to history-freeness of winning strategies in parity games. These fixpoint games have exponential size but we show how to extract *polynomial-size* witnesses for winning strategies of Eloise, and use these witnesses to show that any node won by Eloise is also won in the safety game obtained by a universal graph. For the backwards direction, we show that a witness for satisfaction of the safety condition regarding the universal graph induces a winning strategy in the fixpoint game. This proves that universal graphs can be used to compute nested fixpoints of arbitrary functions over finite lattices and hence yields the quasipolynomial upper bound for computation of nested fixpoints. Moreover, we present a progress measure algorithm that uses the nodes of a quasipolynomial universal graph to measure progress and that can be used to efficiently compute nested fixpoints of arbitrary functions over finite lattices.

As an immediate application of these results, we improve known deterministic algorithms for solving *energy parity games* [10], that is, parity games in which edges have additional integer weights and for which the winning condition is a combined parity condition and a (quantitative) positivity condition on the sum of the accumulated weights. Our results also show that the model checking problem for the associated *energy $\mu$-calculus* [2] is in QP. In a similar fashion, we obtain quasipolynomial algorithms for model checking in latticed $\mu$-calculi [7] in which the truth values of formulae are computed over arbitrary finite lattices, and for solving associated latticed parity games [30].

Furthermore, our results improve generic upper complexity bounds on model checking and satisfiability checking in the *coalgebraic $\mu$-calculus* [12], which serves as a generic framework for fixpoint logics beyond relational semantics. Well-known instances of the coalgebraic $\mu$-calculus include the alternating-time $\mu$-calculus [1], the graded $\mu$-calculus [32], the (two-valued) probabilistic $\mu$-calculus [12, 34], and the monotone $\mu$-calculus [18] (the ambient fixpoint logic of concurrent dynamic logic CPDL [39] and Parikh's game logic [37]). This level of generality is achieved by abstracting system types as set functors and systems as coalgebras for the given functor following the paradigm of universal coalgebra [40]. It was previously shown [24] that the model checking problem for coalgebraic $\mu$-calculi reduces to the computation of a nested fixpoint. This fixpoint may be seen as a coalgebraic generalization of a parity game winning region but can be literally phrased in terms of small standard parity games (implying quasipolynomial run time) only in restricted cases. Our results show that the relevant nested fixpoint can be computed in quasipolynomial time in all cases of interest. Notably, we thus obtain as new specific upper bounds that even under binary coding of numbers, the model checking problems of both the graded $\mu$-calculus and the probabilistic $\mu$-calculus are in QP, even when the syntax is extended to allow for (monotone) polynomial inequalities.

Similarly, the satisfiability problem of the coalgebraic $\mu$-calculus has been reduced to a computation of a nested fixpoint [25], and our present results imply a marked improvement in the exponent of the associated exponential time bound. Specifically, the nesting depth of the relevant fixpoint is exponentially smaller than the basis of the lattice. Our results imply that this fixpoint is computable in polynomial time so that the complexity of satisfiability checking in coalgebraic $\mu$-calculi drops from $2^{\mathcal{O}(n^2k^2 \log n)}$ to $2^{\mathcal{O}(nk \log n)}$ for formulae of size $n$ and with alternation depth $k$.

*Related Work* The quasipolynomial bound on parity game solving has in the meantime been realized by a number of alternative algorithms. For instance, Jurdzinski and Lazic [28] use succinct progress measures to improve to quasilinear (instead of quasipolynomial) space; Fearnley et al. [19] similarly achieve quasilinear space. Lehtinen [33] and Boker and Lehtinen [6] present a quasipolynomial algorithm using register games. Parys [38] improves Zielonka's algorithm [43] to run in quasipolynomial time. In particular the last algorithm is of interest as an additional candidate for generalization to nested fixpoints, due to the known good performance of Zielonka's algorithm in practice. Daviaud et al. [15] generalize quasipolynomial-time parity game solving by providing a pseudo-quasipolynomial algorithm for mean-payoff parity games. On the other hand, Czerwinski et al. [14] give a quasipolynomial lower bound on universal trees, implying a barrier for prospective polynomial-time parity game solving algorithms. Chatterjee et al. [11] describe a quasipolynomial time set-based symbolic algorithm for parity game solving that is parametric in a *lift* function that determines how ranks of nodes depend on the ranks of their successors, and thereby unifies the complexity and correctness analysis of various parity game algorithms. Although part of the parity game structure is encapsulated in a set operator *CPre*, the development is tied to standard parity games, e.g. in the definition of the *best* function, which picks minimal or maximal ranks of successors depending on whether a node belongs to Abelard or Eloise.

Early work on the computation of unrestricted nested fixpoints has shown that greatest fixpoints require less effort in the fixpoint iteration algorithm, which can hence be optimized to compute nested fixpoints with just $\mathcal{O}(n^{\frac{k}{2}})$ calls of the functions at hand [35,41], improving the previously known (straightforward) bound $\mathcal{O}(n^k)$; here, $n$ denotes the size of the basis of the lattice and $k$ the number of fixpoint operators. Recent progress in the field has established the above-mentioned approaches using progress measures [22] and fixpoint games [4] in general settings, both with a view to applications in coalgebraic model checking like in the present paper. In comparison to the present work, the respective bounds on the required number of function iterations in the above unrestricted approaches all are exponential.

A preprint of our present results, specifically the quasipolynomial upper bound on function iteration in fixpoint computation, has been available as an arXiv preprint for some time [23]. Subsequent to this preprint, Arnold, Niwinski and Parys [3] have improved the actual run time by reducing the overhead incurred per iteration (and they give a form of quasipolynomial lower bound for universal-tree-based algorithms), working (like [23]) in the less general setting of directly nested fixpoints over powerset lattices; we show in Section 6 how such an improvement can be incorporated also in our lattice-based algorithm.

## 2 Notation and Preliminaries

Let $U$ and $V$ be sets, and let $R \subseteq U \times U$ be a binary relation on $U$. For $u \in U$, we then put $R(u) := \{v \in U \mid (u,v) \in R\}$. We put $[k] = \{0,\dots,k\}$ for $k \in \mathbb{N}$. *Labelled graphs* $G = (W,R)$ consist of a set $W$ together with a relation $R \subseteq W \times A \times W$ where $A$ is some set of labels; typically, we use $A = [k]$ for some $k \in \mathbb{N}$. An *R-path* in a labelled graph is a finite or infinite sequence $v_0, a_0, v_1, a_1, v_2 \dots$ (ending in a node from $W$ if finite) such that $(v_i, a_i, v_{i+1}) \in R$ for all $i$. For $v \in W$ and $a \in A$, we put $R_a(v) = \{w \in W \mid (v,a,w) \in R\}$ and sometimes write $|G|$ to refer to $|W|$. As usual, we write $U^*$ and $U^\omega$ for the sets of finite sequences or infinite sequences, respectively, of elements of $U$. The *domain* $\mathsf{dom}(f)$ of a partial function $f : U \rightharpoonup V$ is the set of elements on which $f$ is defined. As usual, the *(forward) image* of $A' \subseteq A$ under a function $f : A \to B$ is $f[A'] = \{b \in B \mid \exists a \in A'.\, f(a) = b\}$ and the *preimage* $f^{-1}[B']$ of $B' \subseteq B$ under $f$ is defined by $f^{-1}[B'] = \{a \in A \mid \exists b \in B'.\, f(a) = b\}$. *Projections* $\pi_j : A_1 \times \ldots \times A_m \to A_j$ for $1 \leq j \leq m$ are given by $\pi_i(a_1,\dots,a_m) = a_j$. We often regard (finite) sequences $\tau = u_0, u_1, \ldots \in U^* \cup U^\omega$ of elements of $U$ as partial functions of type $\mathbb{N} \rightharpoonup U$ and then write $\tau(i)$ to denote the element $u_i$, for $i \in \mathsf{dom}(\tau)$. For $\tau \in U^* \cup U^\omega$, we define the set $\mathsf{Inf}(\tau) = \{u \in U \mid \forall i \geq 0.\, \exists j > i.\, \tau(j) = u\}$ of elements that occur infinitely often in $\tau$ (so $\mathsf{Inf}(\tau) = \emptyset$ for $\tau \in U^*$). An infinite $R$-path $v_0, p_0, v_1, p_1, \dots$ in a labelled graph $G = (W,R)$ with labels from $[k]$ is *even* if $\max(\mathsf{Inf}(p_0, p_1, \dots))$ is even, and $G$ is *even* if every infinite $R$-path in $G$ is even. We write $\mathcal{P}(U)$ for the powerset of $U$, and $U^m$ for the $m$-fold Cartesian product $U \times \cdots \times U$.

**Finite Lattices and Fixpoints** A *finite lattice* $(L, \sqsubseteq)$ (often written just as $L$) consists of a non-empty finite set $L$ together with a partial order $\sqsubseteq$ on $L$, such that there is, for all subsets $X \subseteq L$, a join $\bigsqcup X$ and a meet $\bigsqcap X$. The least and greatest elements of $L$ are defined as $\top = \bigsqcup \emptyset$ and element $\top = \bigsqcap \emptyset$, respectively. A set $B_L \subseteq L$ such that $l = \bigsqcup\{b \in B_L \mid b \sqsubseteq l\}$ is a *basis* of $L$. Given a finite lattice $L$, a function $g : L^k \to L$ is *monotone* if $g(V_1,\dots,V_k) \sqsubseteq g(W_1,\dots,W_k)$ whenever $V_i \sqsubseteq W_i$ for all $1 \leq i \leq k$. For monotone $f : L \to L$, we put

$$\mathsf{GFP}\, f = \bigsqcup\{V \sqsubseteq L \mid V \sqsubseteq f(V)\} \qquad \mathsf{LFP}\, f = \bigsqcap\{V \sqsubseteq L \mid f(V) \sqsubseteq V\},$$

which, by the Knaster-Tarski fixpoint theorem, are the greatest and the least fixpoint of $f$, respectively. Furthermore, we define $f^0(V) = V$ and $f^{m+1}(V) = f(f^m(V))$ for $m \geq 0$, $V \sqsubseteq L$; since $L$ is finite, we have $\mathsf{GFP}\, f = f^n(\top)$ and $\mathsf{LFP}\, f = f^n(\bot)$ by Kleene's fixpoint theorem. Given a finite set $U$ and a natural number $n$, $(n^U, \sqsubseteq)$ is a finite lattice, where $n^U = \{f : U \to [n-1]\}$ denotes the function space from $U$ to $[n-1]$ and $f \sqsubseteq g$ if and only if for all $u \in U$, $f(u) \leq g(u)$. For $n = 2$, we obtain the powerset lattice $(2^U, \subseteq)$, also denoted by $\mathcal{P}(U)$, with least and greatest elements $\emptyset$ and $U$, respectively, and basis $\{\{u\} \mid u \in U\}$.

**Parity games** A *parity game* $(V, E, \Omega)$ consists of a set of *nodes* $V$, a left-total relation $E \subseteq V \times V$ of *moves* encoding the rules of the game, and a *priority function* $\Omega : V \to \mathbb{N}$, which assigns *priorities* $\Omega(v) \in \mathbb{N}$ to nodes $v \in V$. Moreover, each node belongs to exactly one of the two players Eloise or Abelard, where we denote the set of Eloise's nodes by $V_\exists$ and that of Abelard's nodes by $V_\forall$. A *play* $\rho \in V^\omega$ is an infinite sequence of nodes that follows the rules of the game, that is, such that for all $i \geq 0$, we have $(\rho(i), \rho(i+1)) \in E$. We say that an infinite play $\rho = v_0, v_1, \ldots$ is *even* if the largest priority that occurs infinitely often in it (i.e. $\max(\mathsf{Inf}(\Omega \circ \rho))$) is even, and *odd* otherwise, and call this property the *parity* of $\rho$. Player Eloise *wins* exactly the even plays and player Abelard *wins* all other plays. A *(history-free)* Eloise-*strategy* $s : V_\exists \rightharpoonup V$ is a partial function that assigns single moves $s(x)$ to Eloise-nodes $x \in \mathsf{dom}(s)$. Given an Eloise-strategy $s$, a play $\rho$ is an *$s$-play* if for all $i \in \mathsf{dom}(\rho)$ such that $\rho(i) \in V_\exists$, we have $\rho(i+1) = s(\rho(i))$. An Eloise-strategy *wins* a node $v \in V$ if Eloise wins all $s$-plays that start at $v$. We have a dual notion of Abelard-strategies; *solving* a parity game consists in computing the *winning regions* $\mathsf{win}_\exists$ and $\mathsf{win}_\forall$ of the two players, that is, the sets of states that they respectively win by some strategy.

It is known that solving parity games is in NP ∩ coNP (and, more specifically, in UP ∩ co-UP). Recently it has also been shown [9] that for parity games with $n$ nodes and $k$ priorities, $\mathsf{win}_\exists$ and $\mathsf{win}_\forall$ can be computed in quasipolynomial time $\mathcal{O}(n^{\log k+6})$. Another crucial property of parity games is that they are *history-free determined* [21], that is, that every node in a parity game is won by exactly one of the two players and then there is a history-free strategy for the respective player that wins the node.

## 3 Systems of Fixpoint Equations

We now introduce our central notion, that is, systems of fixpoint equations over a finite lattice. Throughout, we fix a finite lattice $(L, \sqsubseteq)$ and a basis $B_L$ of $L$ such that $\bot \notin B_L$, and $k+1$ monotone functions $f_i : L^{k+1} \to L$, $0 \leq i \leq k$.

**Definition 3.1.** A *system of equations* consists of $k+1$ equations of the form

$$X_i =_{\eta_i} f_i(X_0, \ldots, X_k)$$

where $\eta_i \in \{\mathsf{LFP}, \mathsf{GFP}\}$, briefly referred to as $f$. For a partial valuation $\sigma : [k] \rightharpoonup L$, we inductively define

$$[\![X_i]\!]^\sigma = \eta_i X_i . f_i^\sigma,$$

where the function $f_i^\sigma$ is given by

$$f_i^\sigma(A) = f_i([\![X_0]\!]^{\sigma'}, \dots, [\![X_{i-1}]\!]^{\sigma'}, A, \mathsf{ev}(\sigma', i+1), \dots, \mathsf{ev}(\sigma', k))$$

for $A \in L$, where $(\sigma[i \mapsto A])(j) = \sigma(j)$ for $j \neq i$ and $(\sigma[i \mapsto A])(i) = A$, $\sigma' = \sigma[i \mapsto A]$ and where $\mathsf{ev}(\sigma, j) = \sigma(j)$ if $j \in \mathsf{dom}(\sigma)$ and $\mathsf{ev}(\sigma, j) = [\![X_j]\!]^\sigma$ otherwise (the latter clause handles *free variables*). Then, the *solution* of the system of equations is $[\![X_k]\!]^\epsilon$ where $\epsilon : [k] \rightharpoonup L$ denotes the empty valuation (i.e. $\mathsf{dom}(\epsilon) = \emptyset$). Similarly, we can obtain solutions for the other components as $[\![X_i]\!]^\epsilon$ for $0 \leq i < k$; we drop the valuation index if no confusion arises, and sometimes write $[\![X_i]\!]_f$ to make the equation system $f$ explicit. We denote by $\mathsf{E}^{f_0}$ the solution $[\![X_k]\!]$ for the *canonical system of equations* of the particular shape

$$X_i =_{\eta_i} X_{i-1} \qquad\qquad X_0 =_{\mathsf{GFP}} f_0(X_0, \dots, X_k),$$

where $0 < i \leq k$, $\eta_i = \mathsf{LFP}$ for odd $i$ and $\eta_i = \mathsf{GFP}$ for even $i$.

**Example 3.2.** (1) *Parity games and the modal $\mu$-calculus:* Let $(V, E, \Omega)$ be a parity game with priorities 0 to $k$, take $L = \mathcal{P}(V)$, and consider the canonical system of fixpoint equations $\mathsf{E}^{f_\exists}$ for the function $f_\exists \colon \mathcal{P}(V)^{k+1} \to \mathcal{P}(V)$ given by

$$f_\exists(V_0, \dots, V_k) = \{v \in V_\exists \mid E(v) \cap V_{\Omega(v)} \neq \emptyset\} \cup \{v \in V_\forall \mid E(v) \subseteq V_{\Omega(v)}, \}$$

for $(V_0, \dots, V_k) \in \mathcal{P}(V)^{k+1}$. It is well known that $\mathsf{win}_\exists = \mathsf{E}^{f_\exists}$, i.e. parity games can be solved by solving fixpoint equation systems. Intuitively, $v \in f_\exists(V_0, \dots, V_k)$ iff Eloise can enforce that some node in $V_{\Omega(v)}$ is reached in the next step. The nested fixpoint expressed by $\mathsf{E}^{f_\exists}$ (in which least (greatest) fixpoints correspond to odd (even) priorities) is constructed in such a way that Eloise only has to rely infinitely often on an argument $V_i$ for odd $i$ if she can also ensure that some argument $V_j$ for $j > i$ is used infinitely often.

Model checking for the *modal $\mu$-calculus* [29] and solving parity games are linear-time equivalent problems. Formulae of the $\mu$-calculus are evaluated over Kripke frames $(U, R)$ with set of states $U$ and transition relation $R$. Formulae $\phi$ of the $\mu$-calculus can be directly represented as equation systems over the lattice $\mathcal{P}(U)$ by recursively translating $\phi$ to equations, mapping subformulae $\mu X_i . \psi(X_0, \dots, X_k)$ and $\nu X_j . \psi(X_0, \dots, X_k)$ to equations

$$X_i =_\mu \psi(X_0, \dots, X_k) \qquad\qquad X_j =_\nu \chi(X_0, \dots, X_k),$$

and interpreting the modalities $\Diamond$ and $\Box$ by functions

$$f_\Diamond(X) = \{u \in U \mid R(u) \cap X \neq \emptyset\} \qquad f_\Box(X) = \{u \in U \mid R(u) \subseteq X\}$$

The solution of the resulting system of equations then is the truth set of the formula $\phi$, that is, model checking for the model $\mu$-calculus reduces to solving fixpoint equation systems. Furthermore, satisfiability checking for the modal $\mu$-calculus can be reduced to solving so-called *satisfiability games* [20], that is, parity games that are played over the set of states of a determinized parity automaton. These satisfiability games can be expressed as systems of fixpoint equations, where the functions track transitions in the determinized automaton.

(2) *Energy parity games and the energy $\mu$-calculus:* Energy parity games [10] are two-player games played over weighted game arenas $(V, E, w, \Omega)$, where $w : E \to \mathbb{Z}$ assigns integer weights to edges. The winning condition is the combination of a parity condition with a (quantitative) positivity condition on the sum of the accumulated weights. It has been shown [2, 10], that $b = n \cdot d \cdot W$ is a sufficient upper bound on energy level accumulations in energy parity games with $n$ nodes, $k$ priorities and maximum absolute weight $W$. We define a function $f_\exists^{\mathsf{e}} : ((b+1)^V)^{k+1} \to (b+1)^V$ over the finite lattice $(b+1)^V$ (whose elements are functions from $V$ to the set $\{0, \dots, b+1\}$) by putting

$$(f_\exists^{\mathsf{e}}(V_0, \dots, V_k))(v) = \begin{cases} \min(\mathsf{en}(v, V_{\Omega(v)})) & \text{if } v \in V_\exists \\ \max(\mathsf{en}(v, V_{\Omega(v)})) & \text{if } v \in V_\forall, \end{cases}$$

for $(V_0, \dots, V_k) \in ((b+1)^V)^{k+1}$ and $v \in V$, using $\mathsf{en}(v, \sigma)$ as abbreviation for

$$\begin{aligned} \mathsf{en}(v, \sigma) = &\{n \in \{0, \dots, b\} \mid \exists u \in E(v).\, n = \max\{0, \sigma(u) - w(v, u)\}\} \cup \\ &\{b+1 \mid \exists u \in E(v).\, \sigma(u) - w(v, u) > b \text{ or } \sigma(u) > b\}, \end{aligned}$$

where $\sigma : V \to \{0, \dots, b+1\}$. Then it follows from the results of [2] that player Eloise wins a node $v$ in the energy parity game with minimal initial credit $c < b+1$ if $(\mathsf{E}^{f_\exists^{\mathsf{e}}})(v) = c$, that is, if the solution of the canonical equation system over $f_\exists^{\mathsf{e}}$ maps $v$ to a value $c$ that is at most $b$.

The *energy $\mu$-calculus* [2] is the fixpoint logic that corresponds to energy parity games. Its formulae are evaluated over *weighted game structures* and involve operators $\Diamond_E \phi$ and $\Box_E \phi$ that are evaluated depending on the energy function $[\![\phi]\!] : V \to \{0, \dots, b+1\}$ that is obtained by first evaluating the argument formula $\phi$. The semantics of the diamond operator then is an energy function that assigns, to each state $v$, the least energy value $c \in \{0, \dots, b+1\}$ such that there is a move from $v$ to some node $u$ such that the credit $c$ suffices to take the move from $v$ to $u$ and retain an energy level of at least $[\![\phi]\!](u)$. Formulae can be translated to equation systems over the finite lattice $(b+1)^V$, where the functions for modal operators are defined according to their semantics as presented in [2]. Solving these equation systems then amounts to model checking energy $\mu$-calculus formulae over weighted game structures.

(3) *Latticed $\mu$-calculi:* In latticed $\mu$-calculi [7], formulae are evaluated over complete lattices $L$ rather than the powerset lattice; for finite lattices $L$, formulae of latticed $\mu$-calculi hence can be translated to fixpoint equation systems over $L$, so that model checking reduces to solving equation systems. An associated latticed variant of games has been introduced in [30] and for finite lattices $L$, solving latticed parity games over $L$ reduces to solving equation systems over $L$.

(4) *The coalgebraic $\mu$-calculus and coalgebraic parity games:* The coalgebraic $\mu$-calculus [12] supports generalized modal branching types by using *predicate liftings* to interpret formulae over $T$-coalgebras, that is, over structures whose transition type is specified by an endofunctor $T$ on the category of sets. For instance the functors $T = \mathcal{P}$, $T = \mathcal{D}$ and $T = \mathcal{G}$ map sets $X$ to their powerset $\mathcal{P}(X)$, the set of probability distributions $\mathcal{D}(X) = \{f : X \to [0, \dots, 1]\}$ over $X$, and to the set of multisets $\mathcal{G}(X) = \{f : X \to \mathbb{N}\}$ over $X$, respectively. The corresponding $T$-coalgebras then are Kripke frames (for $T = \mathcal{P}$), Markov chains (for $T = \mathcal{D}$) and graded transition systems (for $T = \mathcal{G}$), respectively. Instances of the coalgebraic $\mu$-calculus comprise, e.g. the two-valued probabilistic $\mu$-calculus [12, 34] with modalities $\Diamond_p \phi$ for $p \in [0, \dots, 1]$, expressing 'the next state satisfies $\phi$ with probability more than $p$'; the graded $\mu$-calculus [32] with modalities $\Diamond_g \phi$ for $g \in \mathbb{N}$, expressing 'there are more than $\phi$ successor states that satisfy $\phi$'; or the alternating-time $\mu$-calculus [1] that is interpreted over concurrent game frames and uses modalities $\langle D \rangle \phi$ for finite $D \subseteq \mathbb{N}$ (encoding a *coalition*) that express that 'coalition $D$ has a joint strategy to enforce $\phi$'.

It has been shown in previous work [24] that model checking for coalgebraic $\mu$-calculi against coalgebras with state space $U$ reduces to solving a canonical fixpoint equation system over the powerset lattice $\mathcal{P}(U)$, where the involved function interprets modal operators using predicate liftings, as described in [12, 24]. This canonical equation system can alternatively be seen as the winning region of Eloise in *coalgebraic parity games*, a highly general variant of parity games where the game structure is a coalgebra and nodes are annotated with modalities. Examples include *two-valued probabilistic parity games* and *graded parity games* in which nodes and edges are annotated with probabilities or grades, respectively. In order to win a node $v$, player Eloise then has to have a strategy that picks a *set* of moves to nodes that in turn are *all* won by Eloise, and such that the joint probability (joint grade) of the picked moves is greater than the probability (grade) that is assigned to $v$. It is known that solving coalgebraic parity games reduces to solving fixpoint equation systems [24].

Furthermore, the satisfiability problem of the coalgebraic $\mu$-calculus has been reduced to solving canonical fixpoint equations systems over lattices $\mathcal{P}(U)$, where $U$ is the state set of a determinized parity automaton and where the innermost equation checks for joint one-step satisfiability of sets of coalgebraic modalities [25]. By interpreting coalgebraic formulae over finite lattices $d^U$ rather than over powerset lattices, one obtains the *finite-valued coalgebraic $\mu$-calculus* (with values $\{0, \dots, d\}$), which has the *finite-valued probabilistic $\mu$-calculus* (e.g. [36]) as an instance. Model checking for the finite-valued probabilistic $\mu$-calculus hence reduces to solving equation systems over the finite lattice $d^{|U|}$, where $\{0, \dots, d\}$ encodes a finite set of probabilities.

## 4 Fixpoint Games and History-free Witnesses

We instantiate the existing notion of fixpoint games [4, 42], which characterize solutions of equation systems, to our setting (that is, to finite lattices), and then use these games as a technical tool to establish our crucial notion of history-freeness for systems of fixpoint equations.

**Definition 4.1 (Fixpoint games).** Let $X_i =_{\eta_i} f_i(X_0, \ldots, X_k)$, $0 \le i \le k$, be a system of fixpoint equations. The associated *fixpoint game* is a parity game $(V, E, \Omega)$ with set of nodes $V = (B_L \times [k]) \cup L^{k+1}$, where nodes from $B_L \times [k]$ belong to player $\mathsf{Eloise}$ and nodes from $L^{k+1}$ belong to player $\mathsf{Abelard}$. For nodes $(u, i) \in B_L \times [k]$, we put

$$E(u, i) = \{(U_0, \ldots, U_k) \in L^{k+1} \mid u \sqsubseteq f_i(U_0, \ldots, U_k)\},$$

and for nodes $(U_0, \ldots, U_k) \in L^{k+1}$, we put

$$E(U_0, \ldots, U_k) = \{(u, i) \in B_L \times [k] \mid u \sqsubseteq U_i\}.$$

The *alternation depth* $\mathsf{ad}(i)$ of an equation $X_i =_{\eta_i} f_i(X_0, \ldots, X_1)$ is defined as $\mathsf{ad}_i^\mu$ if $\eta_i = \mu$ and as $\mathsf{ad}_i^\nu$ if $\eta_i = \nu$, where $\mathsf{ad}_i^\mu$, $\mathsf{ad}_i^\nu$ are recursively defined by

$$\mathsf{ad}_i^\mu = \begin{cases} \mathsf{ad}_{i-1}^\mu & i > 0, \eta_{i-1} = \mu \\ \mathsf{ad}_{i-1}^\nu + 1 & i > 0, \eta_{i-1} = \nu \\ 1 & i = 0 \end{cases} \qquad \mathsf{ad}_i^\nu = \begin{cases} \mathsf{ad}_{i-1}^\mu + 1 & i > 0, \eta_{i-1} = \mu \\ \mathsf{ad}_{i-1}^\nu & i > 0, \eta_{i-1} = \nu \\ 0 & i = 0 \end{cases}$$

for $0 \le i \le k$. The priority function $\Omega : V \to [\mathsf{ad}(k)]$ then is defined by $\Omega(u, i) = \mathsf{ad}(i)$ and $\Omega(U_0, \ldots, U_k) = 0$.

**Remark 4.2.** In [4], an alternative priority function $\Omega' : V \to [2k + 1]$ with

$$\Omega'(u, i) = \begin{cases} 2i & \text{if } \eta_i = \mathsf{GFP} \\ 2i + 1 & \text{if } \eta_i = \mathsf{LFP} \end{cases}$$

and $\Omega'(U_0, \ldots, U_k) = 0$ is used. Since $\mathsf{ad}(i)$ is even if and only if $\eta_i$ is even, and moreover $\mathsf{ad}(\mathsf{i}) \le \mathsf{ad}(j)$ for $i \le j$, and $i < j$ whenever $\mathsf{ad}(\mathsf{i}) < \mathsf{ad}(j)$, it is easy to see that $\Omega$ and $\Omega'$ in fact assign identical parities to all plays. In the following, we will use the more economic parity function $\Omega$ so that fixpoint games have only $d := \mathsf{ad}(k) \le k$ priorities.

We import the associated characterization theorem [4, Theorem 4.8]:

**Theorem 4.3 ([4]).** *We have $u \sqsubseteq [\![X_i]\!]_f$ if and only if* $\mathsf{Eloise}$ *wins the node $(u, i)$ in the fixpoint game for the given system $f$ of equations.*

**Remark 4.4.** While this shows that parity game solving can be used to solve equation systems, the size of fixpoint games is exponential in $|B_L|$, so they do not directly yield a quasipolynomial algorithm for solving equation systems.

Next we define our notion of history-freeness for systems of fixpoint equations.

**Definition 4.5 (History-free witness).** A *history-free witness* for $u \sqsubseteq [\![X_i]\!]_f$ is an even labelled graph $(W, R)$ with labels from $[d]$ such that $W \subseteq B_L \times [d]$, $(u, i) \in W$, and for all $(v, p) \in W$, we have $v \sqsubseteq f_p(U_0, \ldots, U_k)$ where $U_j = \bigsqcup \pi_1[R_{\mathsf{ad}(j)}(v, p)]$ for $0 \leq j \leq k$, noting that $R_{\mathsf{ad}(j)}(v, p) \subseteq W$ so that $\pi_1[R_{\mathsf{ad}(j)}(v, p)] \subseteq B_L$ and $U_j \in L$.

In analogy to history-free strategies for parity games, history-free witnesses assign tuples $(R_1(v, p), \ldots, R_d(v, p))$ of sets $R_j(v, p) \subseteq W$ to pairs $(v, p) \in W$ without relying on a history of previously visited pairs. We have $|W| \leq (d+1)|B_L|$ and $|R| \leq (d+1)|W|^2$, that is, the size of history-free witnesses is polynomial in $|B_L|$. Crucially, history-free witnesses always exist:

**Lemma 4.6.** *For all $u \in B_L$ and $i \in [k]$, we have*

$$u \sqsubseteq [\![X_i]\!]_f \text{ if and only if there is a history-free witness for } u \sqsubseteq [\![X_i]\!]_f.$$

*Proof.* In one direction, we have $u \sqsubseteq [\![X_i]\!]_f$ so that Eloise wins the node $(u, i)$ in the according fixpoint game by Lemma 4.3. Let $s$ be a corresponding *history-free* winning strategy (such strategies always exists, see e.g. [21]). We inductively construct a witness for $u \sqsubseteq [\![X_i]\!]_f$, starting at $(u, i)$. When at $(v, p) \in B_L \times [k]$ with $s(v, p) = (U_0, \ldots, U_k)$, we put $R_i(v, p) = \bigcup_{j \mid \mathsf{ad}(j) = i}(U_j \times \{j\})$ for $0 \leq i \leq d$ and hence have $\mathsf{ad}(j) = i$ for all $((v, p), i, (u, j)) \in R$. Since $s$ is a winning strategy, the resulting graph $(W, R)$ is a history-free witness for $u \sqsubseteq [\![X_i]\!]_f$ by construction; in particular, $(W, R)$ is even. For the converse direction, the witness for $u \sqsubseteq [\![X_i]\!]_f$ directly yields a winning Eloise-strategy for the node $(u, i)$ in the associated fixpoint game. This implies $u \sqsubseteq [\![X_i]\!]_f$ by Lemma 4.3. □

## 5 Solving Equation Systems using Universal Graphs

We go on to prove our main result. To this end, we fix a system $f$ of fixpoint equations $f_i : L^{k+1} \to L$, $0 \leq i \leq k$, and put $n := |B_L|$ and $d := \mathsf{ad}(k)$ for the remainder of the paper.

**Definition 5.1 (Universal graphs [13, 14]).** Let $G = (W, R)$ and $G' = (W', R')$ be labelled graphs with labels from $[d]$. A *homomorphism of labelled graphs* from $G$ to $G'$ is a function $\Phi : W \to W'$ such that for all $(v, p, w) \in R$, we have $(\Phi(v), p, \Phi(w)) \in R'$. An $(n, d+1)$*-universal graph* $S$ is an even graph with labels from $[d]$ such that for all even graphs $G$ with labels from $[d]$ and with $|G| \leq n$, there is a homomorphism from $G$ to $S$.

We fix an $(n(d+1), (d+1))$-universal graph $S = (Z, K)$, noting that there are $(n(d+1), (d+1))$-universal graphs (obtained from universal trees) of size quasipolynomial in $n$ and $d$ [14]. We now combine the system $f$ with the universal graph $S$ to turn the parity conditions associated to general systems of fixpoint equations into a safety condition, associated to a single greatest fixpoint equation.

**Definition 5.2 (Chained-product fixpoint).** We define a function

$$\begin{aligned} g\colon \mathcal{P}(B_L \times [k] \times Z) &\to \mathcal{P}(B_L \times [k] \times Z) \\ U \quad &\mapsto \{(v,p,q) \in B_L \times [k] \times Z \mid v \sqsubseteq f_p(P_0^{U,q},\dots,P_k^{U,q})\} \end{aligned}$$

where

$$P_i^{U,q} = \bigsqcup \{u \in B_L \mid \exists s \in K_{\mathsf{ad}(i)}(q).\,(u,i,s) \in U\}.$$

We refer to $Y_0 =_{\mathsf{GFP}} g(Y_0)$ as the *chained-product fixpoint (equation)* of $f$ and $S$.

We now show our central result: apart from the annotation with states from the universal graph, the chained-product fixpoint $g$ is the solution of the system $f$.

**Theorem 5.3.** *For all $u \in B_L$ and $0 \le i \le k$, we have*

$$u \sqsubseteq [\![X_i]\!]_f \text{ if and only if there is } q \in Z \text{ such that } (u,i,q) \in [\![Y_0]\!]_g.$$

*Proof.* For the forward direction, let $u \sqsubseteq [\![X_i]\!]_f$. By Lemma 4.6, there is a history-free witness $G = (W,R)$ for $u \sqsubseteq [\![X_i]\!]_f$. Since $S$ is a $(n(d+1), d+1)$-universal graph and since $G$ is a witness and hence an even labelled graph of suitable size $|G| \le n(d+1)$, there is a graph homomorphism $\Phi$ from $G$ to $S$. Starting at $(u,i,\Phi(u,i),0)$, we inductively construct a witness for containment of $(u,i,\Phi(u,i))$ in $[\![Y_0]\!]_g$. When at $(v_1,p_1,\Phi(v_1,p_1),0)$ with $(v_1,p_1) \in W$, we put

$$\begin{aligned} R'_0(v_1,p_1,\Phi(v_1,p_1),0) =& \{(v_2,p_2,\Phi(v_2,p_2),0) \in B_L \times [d] \times Z \times [0] \mid \\ & (v_2,p_2) \in R_{\mathsf{ad}(p_2)}(v_1,p_1), \Phi(v_2,p_2) \in K_{\mathsf{ad}(p_2)}(\Phi(v_1,p_1))\,\} \end{aligned}$$

and continue the inductive construction with all these $(v_2,p_2,\Phi(v_2,p_2),0)$, having $(v_2,p_2) \in W$. The resulting structure $G' = (W',R')$ indeed is a witness for containment of $(u,i,q)$ in $[\![Y_0]\!]_g$: $G'$ is even by construction. Moreover, we need to show that for $(v_1,p_1,\Phi(v_1,p_1),0) \in W'$, we have $(v_1,p_1,\Phi(v_1,p_1),0) \in g(\pi_1[R'_0(v_1,p_1,\Phi(v_1,p_1),0)])$, i.e. $v_1 \sqsubseteq f_{p_1}(P_0^{U,\Phi(v_1,p_1)},\dots,P_k^{U,\Phi(v_1,p_1)})$ where $U = \pi_1[R'_0(v_1,p_1,\Phi(v_1,p_1),0)]$. Since $G$ is a witness and $(v_1,p_1) \in W$ by construction of $W'$, we have $v_1 \sqsubseteq f_{p_1}(U_0,\dots,U_k)$ where $U_j = \bigsqcup(\pi_j[R_{\mathsf{ad}(i)}(v_1,p_1)])$. By monotonicity of $f_{p_1}$, it thus suffices to show that $U_j \sqsubseteq P_j^{U,\Phi(v_1,p_1)}$ for $0 \le j \le k$; by definition of $P_j^{U,\Phi(v_1,p_1)}$ this follows if

$$\pi_1[R_{\mathsf{ad}(j)}(v_1,p_1)] \subseteq \{u \in B_L \mid \exists s \in K_{\mathsf{ad}(j)}(\Phi(v_1,p_1)).(u,j,s) \in W\},$$

where $W = \pi_1[R'_0(v_1,p_1,q_1,0)]$. So let $w \in B_L$ such that $w \in \pi_1[R_{\mathsf{ad}(j)}(v_1,p_1)]$. Since $R$ is a witness that is constructed as in the proof of Lemma 4.6, we have $i = \mathsf{ad}(i')$ for all $((v',p'),i,(w',i')) \in R$. Thus $(w,j) \in R_{\mathsf{ad}(j)}(v_1,p_1)$ for some $j$ such that $\mathsf{ad}(j) = i$, that is, $((v_1,p_1),\mathsf{ad}(j),(w,j)) \in R$, hence $(\Phi(v_1,p_1),\mathsf{ad}(j),\Phi(w,j)) \in K$ because $\Phi$ is a graph homomorphism. By definition of $R'_0$ we have $(w,j,\Phi(w,j),0) \in R'_0(v_1,p_1,\Phi(v_1,p_1),0)$ so that $(w,j,\Phi(w,j)) \in \pi_1[R'_0(v_1,p_1,\Phi(v_1,p_1),0)]$. We are done since $\Phi(w,j) \in K_{\mathsf{ad}(j)}(\Phi(v_1,p_1))$.

For the converse implication, let $(u_0, p_0, q_0) \in [\![Y_0]\!]_g$ for some $q_0 \in Z$. Let $G = (W, R)$ be a history-free witness for this fact. By Lemma 4.3, it suffices to provide a strategy in the fixpoint game for the system $f$ with which Eloise wins the node $(u_0, p_0)$. We inductively construct a *history-dependent* strategy $s$ as follows: For $i \geq 0$, we abbreviate $U_i = R_0(u_i, p_i, q_i, 0)$. We put $s(u_0, p_0) = (P_0^{U_0,q_0}, \ldots, P_k^{U_0,q_0})$. For the inductive step, let

$$\tau = (u_0, p_0), (P_0^{U_0,q_0}, \ldots, P_k^{U_0,q_0}), \ldots, (P_0^{U_{n-1},q_{n-1}}, \ldots, P_k^{U_{n-1},q_{n-1}}), (u_n, p_n)$$

be a partial play of the fixpoint game that follows the strategy that has been constructed so far. Then we have an $R$-path $(u_0, p_0, q_0, 0), (u_1, p_1, q_1, 0), \ldots, (u_n, p_n, q_n, 0)$, where, for $0 \leq i < n$, we have $(q_i, p_{i+1}, q_{i+1}) \in K$ since $u_{i+1} \sqsubseteq P_{p_{i+1}}^{U_i,q_i}$ by the inductive construction. Put $s(\tau) = (P_0^{U_n,q_n}, \ldots, P_k^{U_n,q_n})$. Since $G$ is a witness, the strategy uses only moves that are available to Eloise (i.e. ones with $u_n \sqsubseteq f_{p_n}(s(\tau))$). Also, $s$ is a winning strategy as can be seen by looking at the $K$-paths that are induced by complete plays $\tau$ that follow $s$, as described (for partial plays) above. Since $S$ is a universal graph and hence even, every such $K$-path is even and the sequence of priorities in $\tau$ is just the sequence of priorities of one of these $K$-paths. □

**Remark 5.4.** Since the set $[\![Y_0]\!]_g$ is the greatest fixpoint of $g$, it can be computed by simple approximation from above, that is, as $g^m(B_L \times [k] \times Z)$ where $m = |B_L \times [k] \times Z|$. However, each iteration of the function $g$ may require up to $|Z|$ evaluations of an equation. In the next section, we will show how this additional iteration factor in the computation of $[\![Y_0]\!]_g$ can be avoided.

## 6 A Progress Measure Algorithm

We next introduce a lifting algorithm that computes the set $[\![Y_0]\!]_g$ efficiently, following the paradigm of the progress measure approach for parity games (e.g. [27,28]). Our progress measures will map pairs $(u, i) \in B_L \times [k]$ to nodes in a universal graph that is equipped with a simulation order, that is, a total order that is suitable for measuring progress.

**Definition 6.1 (Simulation order).** For natural numbers $i$, $i'$, we put $i \succeq i'$ if and only if either $i$ is even and $i = i'$, or both $i$ and $i'$ are odd and $i \geq i'$. A total order $\leq$ on $Z$ is a *simulation order* if for all $q, q' \in Z$,

$$q \leq q' \text{ implies that for all } 0 \leq i \leq k \text{ and } s \in K_i(q), \text{ there are } i' \succeq i \text{ and } s' \in K_{i'}(q') \text{ such that } s \leq s'.$$

**Lemma 6.2.** *There is an $(n(d+1), d+1)$-universal graph $(Z, K)$ of size quasipolynomial in $n$ and $d$, and over which a simulation order $\leq$ exists.*

*Proof (Sketch).* It has been shown [14, Theorem 2.2] (originally, in different terminology, [28]) that there are $(l, h)$-*universal trees* (a concept similar to, but slightly more concrete than universal graphs) with set of leaves $T$ such that $|T| \leq 2l\binom{\log l+h+1}{h}$. Leaves in universal trees are identified by *navigation paths*, that is, sequences of branching directions, so that the leaves are linearly ordered by the lexicographic order $\leq$ on navigation paths (which orders leafs from the left to the right). As described in [13], one can obtain a universal graph $(T, K)$ over $T$ in which transitions $(q, i, q') \in K$ for odd $i$ (the crucial case) move to the left, that is, $q'$ is a leaf that is to the left of $q$ in the universal tree (so that $q' < q$), ensuring universality. As it turns out, the lexicographic ordering on $T$ is a simulation order. Adapting this construction to our setting, we put $l = n(d+1)$ and $h = d+1$ and obtain a $(n(d+1), d+1)$-universal graph (along with a simulation order $\leq$) of size at most $2n(d+1)\binom{\log(n(d+1))+d+2}{d+1}$ which is quasipolynomial in $n$ and $d$. □

We fix an $(n(d+1), d+1)$-universal graph $(Z, K)$ and a simulation order $\leq$ on $Z$ for the remainder of the paper (these exist by the above lemma).

**Definition 6.3 (Progress measure, lifting function).** We let $q_{\mathsf{min}} \in Z$ denote the least node w.r.t. $\leq$ and fix a distinguished top element $\star \notin Z$, and extend $\geq$ to $Z \cup \{\star\}$ by putting $\star \geq q$ for all $q \in Z$. A *measure* is a map $\mu\colon B_L \times [k] \to Z \cup \{\star\}$, i.e. assigns nodes in the universal graph or $\star$ to pairs $(v, p) \in B_L \times [k]$. A measure $\mu$ is a *progress measure* if whenever $\mu(v, p) \neq \star$, then $v \sqsubseteq f_p(U_0^{\mu,q}, \dots, U_k^{\mu,q})$ where $q = \mu(v, p)$ and

$$U_i^{\mu,q} = \bigsqcup\{u \in B_L \mid \exists s \in K_{\mathsf{ad}(i)}(q).\, \mu(u, i) \leq s\}.$$

We define a function $\mathsf{Lift} : (B_L \times [k] \to Z \cup \{\star\}) \to (B_L \times [k] \to Z \cup \{\star\})$ on measures by

$$(\mathsf{Lift}(\mu))(v, p) = \min\{q \in Z \mid v \sqsubseteq f_p(U_0^{\mu,q}, \dots, U_k^{\mu,q})\}$$

where $\min(Z')$ denotes the least element of $Z'$ w.r.t. $\leq$, for $\emptyset \neq Z' \subseteq Z$; also we put $\min(\emptyset) = \star$.

The lifting algorithm then starts with the least measure $\mathsf{m}_{\min}$ that maps all pairs $(v, p) \in B_L \times [k]$ to the minimal node (i.e. $\mathsf{m}_{\min}(v, p) = q_{\min}$) and repeatedly updates the current measure using $\mathsf{Lift}$ until the measure stabilizes.

**Lifting algorithm**

(1) Initialize: Put $\mu := \mathsf{m}_{\min}$.

(2) If $\mathsf{Lift}(\mu) \neq \mu$, then put $\mu := \mathsf{Lift}(\mu)$ and go to 2. Otherwise go to 3.

(3) Return the set $\mathbb{E} = \{(v, p) \in B_L \times [k] \mid \mu(v, p) \neq \star\}$.

**Lemma 6.4 (Correctness).** *For all $v \in B_L$ and $0 \leq p \leq k$, we have*

$$(v, p) \in \mathbb{E} \text{ if and only if } v \in [\![X_p]\!]_f.$$

*Proof (Sketch).* Let $\mu$ denote the progress measure that the algorithm computes. For one direction of the proof, let $(v,p) \in \mathbb{E}$. By Lemma 4.6 it suffices to construct a witness for $v \in [\![X_p]\!]_f$. We extract such a witness $(\mathbb{E}, R)$ from the progress measure $\mu$, relying on the properties of the simulation order $\leq$ that is used to measure the progress of $\mu$ to ensure that any infinite sequence of measures that $\mu$ assigns to some $R$-path induces an infinite (and hence even) path in the employed universal graph. This shows that $(\mathbb{E}, R)$ indeed is an even graph and hence a witness. For the converse direction, let $v \in [\![X_p]\!]_f$ so that there is, by Theorem 5.3, some $q \in Z$ such that $(v,p,q) \in [\![Y_0]\!]_g$. For $(u,i)$ such that there is $q' \in Z$ such that $(u,i,q') \in [\![Y_0]\!]_g$, let $q_{(u,i)} \in Z$ denote the minimal such node w.r.t. $\leq$. It now suffices that $\mu(u,i) \leq q_{(u,i)}$ for all such $(u,i)$, which is shown by induction on the number of iterations of the lifting algorithm. □

**Corollary 6.5.** *Solutions of systems of fixpoint equations can be computed with quasipolynomially many evaluations of equations.*

*Proof.* Given an $(n(d+1), d+1)$-universal graph $(Z, K)$ and a simulation order on $Z$, the lifting algorithm terminates and returns the solution of $f$ after at most $n(d+1) \cdot |Z|$ many iterations. This is the case since each iteration (except the final iteration) increases the measure for at least one of the $n(d+1)$ nodes and the measure of each node can be increased at most $|Z|$ times. Using the universal graph and the simulation order from the proof of Lemma 6.2, we have $|Z| \leq 2n(d+1)\binom{\log(n(d+1))+d+2}{d+1}$ so that the algorithm terminates after at most $2(n(d+1))^2\binom{\log(n(d+1))+d+2}{d+1} \in \mathcal{O}((n(d+1))^{\log(d+1)})$ iterations of the function $\mathsf{Lift}$. Each iteration can be implemented to run with at most $n(d+1)$ evaluations of an equation. □

**Corollary 6.6.** *The number of function calls required for the solution of systems of fixpoint equations with $d \leq \log n$ is bounded by a polynomial in $n$ and $d$.*

*Proof.* Following the insight of Theorem 2.8 in [9], Theorem 2.2. in [14] implies that if $d < \log n$, then there is an $(n(d+1), d+1)$-universal tree of size polynomial in $n$ and $d$. In the same way as in the proof of Lemma 6.2, one obtains a universal graph of polynomial size and a simulation order on it. □

**Example 6.7.** Applying Corollary 6.5 and Corollary 6.6 to Example 3.2, we obtain the following results:

(1) The model checking problems for the energy $\mu$-calculus and finite latticed $\mu$-calculi are in QP. For energy parity games with sufficient upper bound $b$ on energy level accumulations, we obtain a progress measure algorithm that terminates after a number of iterations that is quasipolynomial in $b$.

(2) Under mild assumptions on the modalities (see [24]), the model checking problem for the coalgebraic $\mu$-calculus is in QP; in particular, this yields QP model checking algorithms for the graded $\mu$-calculus and the two-valued probabilistic $\mu$-calculus (equivalently: QP progress measure algorithms for solving graded and two-valued probabilistic parity games).

(3) Under mild assumptions on the modalities (see [25]), we obtain a novel upper bound $2^{\mathcal{O}(nd\log n)}$ for the satisfiability problems of coalgebraic $\mu$-calculi, in particular including the monotone $\mu$-calculus, the alternating-time $\mu$-calculus, the graded $\mu$-calculus and the (two-valued) probabilistic $\mu$-calculus, even when the latter two are extended with (monotone) polynomial inequalities. This improves on the best previous bounds in all cases.

## 7 Conclusion

We have shown how to use universal graphs to compute solutions of systems of fixpoint equations $X_i = \eta_i.\, f_i(X_0, \dots, X_k)$ (with the $\eta_i$ marking least or greatest fixpoints) that use functions $f_i : L^{k+1} \to L$ (over a finite lattice $L$ with basis $B_L$) and involve up to $k + 1$-fold nesting of fixpoints. Our progress measure algorithm needs quasipolynomially many evaluations of equations, and runs in time $\mathcal{O}(q \cdot t(f))$, where $q$ is a quasipolynomial in $|B_L|$ and the alternation depth of the equation system, and where $t(f)$ is an upper bound on the time it takes to compute $f_i$ for all $i$.

As a consequence of our results, the upper time bounds for the evaluation of various general parity conditions improve. Example domains beyond solving parity games to which our algorithm can be instantiated comprise model checking for latticed $\mu$-calculi and solving latticed parity games [7, 30], solving energy parity games and model checking for the energy $\mu$-calculus [2, 10], and model checking and satisfiability checking for the coalgebraic $\mu$-calculus [12]. The resulting model checking algorithms for latticed $\mu$-calculi and the energy $\mu$-calculus run in time quasipolynomial in the provided basis of the respective lattice. In terms of concrete instances of the coalgebraic $\mu$-calculus, we obtain, e.g., quasipolynomial-time model checking for the graded [32] and the probabilistic $\mu$-calculus [12, 34] as new results (corresponding results for, e.g., the alternating-time $\mu$-calculus [1] and the monotone $\mu$-calculus [18] follow as well but have already been obtained in our previous work [24]), as well as improved upper bounds for satisfiability checking in the graded $\mu$-calculus, the probabilistic $\mu$-calculus, the monotone $\mu$-calculus, and the alternating-time $\mu$-calculus. We foresee further applications, e.g. in the computation of fair bisimulations and fair equivalence [26, 31] beyond relational systems, e.g. for probabilistic systems.

As in the case of parity games, a natural open question that remains is whether solutions of fixpoint equations can be computed in polynomial time (which would of course imply that parity games can be solved in polynomial time). A more immediate perspective for further investigation is to generalize the recent quasipolynomial variant [38] of Zielonka's algorithm [43] for solving parity games to solving systems of fixpoint equations, with a view to improving efficiency in practice.
